# Are patients and health care professionals influenced by each other's’ opinions about the outcomes to measure in localised prostate cancer effectiveness trials?

**DOI:** 10.1186/1745-6215-16-S2-P64

**Published:** 2015-11-16

**Authors:** Steven MacLennan, Thomas Lam, Paula Williamson

**Affiliations:** 1 University of Aberdeen, Aberdeen, UK; 2 University of Liverpool, Liverpool, UK

## Background

A core outcome set (COS) is required for effectiveness trials in localised prostate cancer due to outcome heterogeneity. The study aims to develop a COS for localised prostate cancer trials, involving a nested randomised controlled trial (RCT) design within a Delphi survey, to explore and understand how patients and clinicians influence each other's decision-making in the survey.

## Methods and design

Two stakeholder groups (patients and clinicians) are included. A three round online Delphi is being used to prioritise a list of outcomes identified in systematic reviews and patient interviews. A methodological sub-study (nested RCT) within the Delphi is also underway. 118 patients and 56 clinicians completed round 1. Participants, stratified by stakeholder group were allocated after round one, using variable block randomisation, to one of three feedback strategies groups. Participants were asked to provide their reasons for changing their scores between rounds.

## Results

The outcome of interest for the nested RCT is the difference between elements of the COS developed in each randomised group. The results of the Delphi are expected to be known by October 2015 and will be presented at the ICTMC. If the trial finds no obvious differences between the approaches, it would not be inferred that they are intrinsically the same or that this finding could be applied in other settings (since it could be due to inadequate power or homogeneity of opinion in the studied area); but if important differences are detected, this may have important implications for future development of core outcome sets.

